# Supplementary material for: Integrated Machine Learning Approach for the Early Prediction of Pressure Ulcers in Spinal Cord Injury Patients
Source: J Clin Med. 2024 Feb 8;13(4):990. doi: 10.3390/jcm13040990 (PMC10889422; doi:10.3390/jcm13040990)
Supplement: Supplementary file 1 [file jcm-13-00990-s001.zip › jcm-2806390-supplementary.pdf]

Supplementary Materials

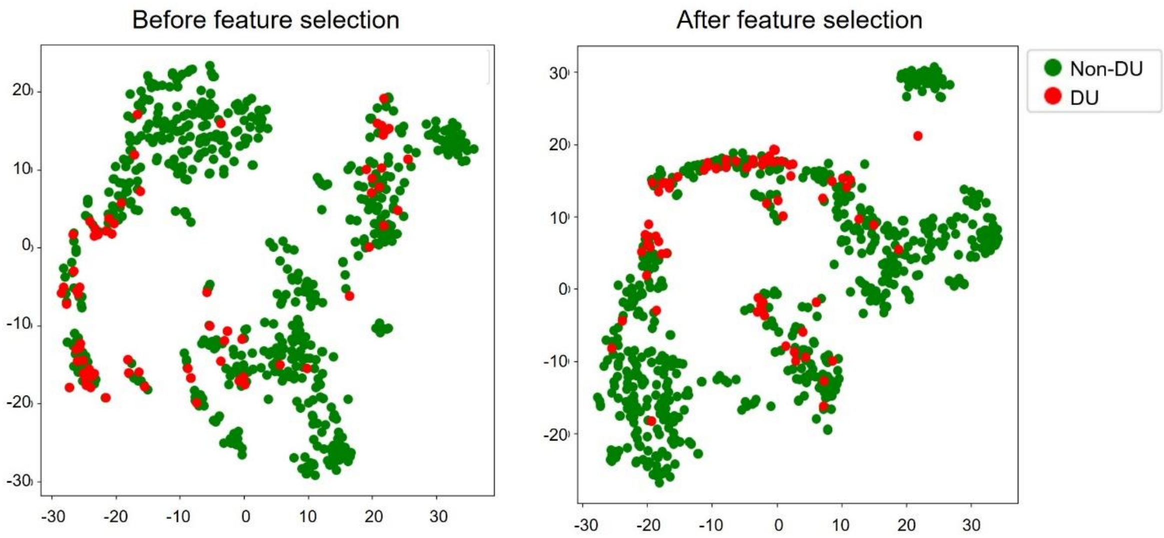

Figure S1. tSNE plot before and after feature selection in CNUH data

The tSNE plot from the KNN model classified PUs more clearly after feature selection.

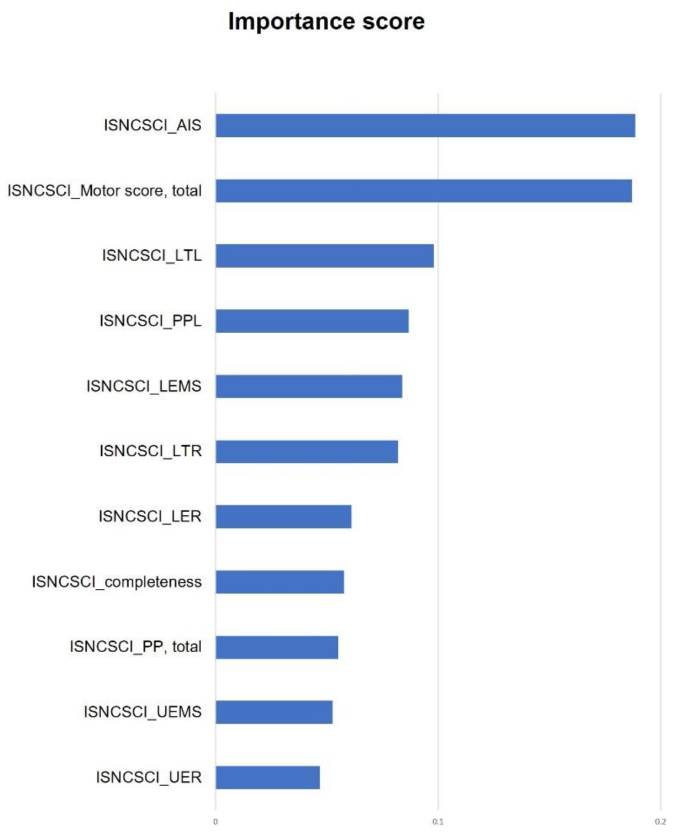

Figure S2. Importance scores of the top 11 featured parameters based on the outcome of the KNN model in CNUH

The top 11 featured parameters were ranked by importance, and the 'AIS' item from the ISNCSCI had the highest importance score.

Abbreviations: ISNCSCI = International Standards for Neurological Classification of Spinal Cord Injury; AIS = ASIA impairment scale; LTL = Light touch; PPL = Pinprick, left; LEMS = Lower Extremity Motor Subscore; LTR = Light touch, right; LER = Lower extremity, right; PP, total = Pinprick, total; UEMS = Upper Extremity Motor Subscore; UER = Upper extremity, right.

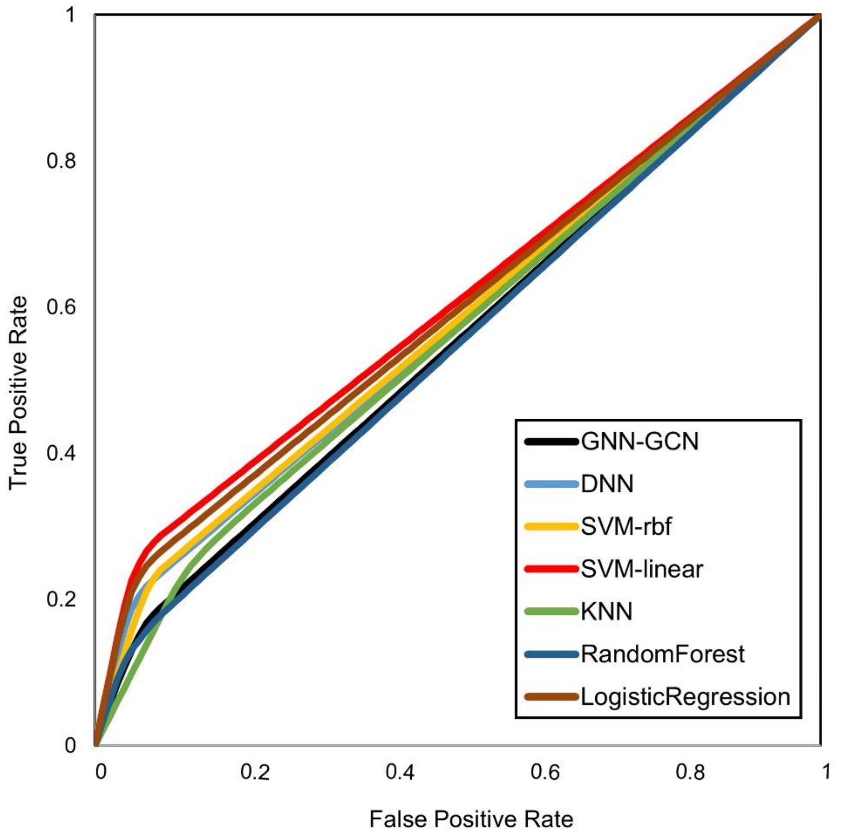

Figure S3. Receiver operating characteristic (ROC) curve of each machine learning algorithm in DKUH using the ISNCSCI dataset
